# Exploring Alzheimer Disease from a Retinal and Ocular Perspective

**DOI:** 10.3390/biomedicines14071465

**Published:** 2026-06-28

**Authors:** Joel Victoria-Martínez, José Martín-Nieto

**Affiliations:** 1 Departamento de Fisiología, Genética y Microbiología, Facultad de Ciencias, Universidad de Alicante, 03080 Alicante, Spain; 2 Instituto de Investigación Sanitaria y Biomédica de Alicante (ISABIAL), 03010 Alicante, Spain; 3 Instituto Multidisciplinar para el Estudio del Medio ‘Ramón Margalef’, Universidad de Alicante, 03080 Alicante, Spain

**Keywords:** Alzheimer disease, eye biomarkers, retinal alterations, ocular impairments, diagnosis, prognosis

## Abstract

Alzheimer disease (AD) is a neurodegenerative disorder currently recognized as the leading cause of dementia worldwide. It is characterized by a progressive cognitive decline, which can be studied and diagnosed through the use of various brain biomarkers. The retina, being part of the central nervous system, shares numerous structural and functional features with the brain. In this light, a wide range of alterations have been found in the retina with significant potential as biomarkers for AD diagnosis, even at early stages of its manifestation in the brain, and for monitoring disease progression within this organ. Furthermore, the detection of such alterations in the eye and retina is feasible through non-invasive, relatively simple and cost-effective techniques, such as optical coherence tomography, scanning laser ophthalmoscopy and electroretinography. Using these methods, numerous studies have identified molecular, morphological and functional changes associated with AD in the retina and other ocular elements, including the choroid, cornea, lens, intraocular humors and tear fluid. This review addresses the main anomalous changes identified to date in the retina and other eye structures in patients with AD, highlighting their potential utility as biomarkers for the diagnosis of this disease and their possible extrapolation to its prognosis in the brain.

## 1. Introduction

Alzheimer disease (AD) is a neurodegenerative disorder and the leading cause of dementia worldwide, currently affecting over 50 million people, and whose prevalence is expected to triple by 2050 [1,2]. It was first described in 1901 by the German neuropsychiatrist Alois Alzheimer, after whom the disease is named [3]. To date, there is no effective treatment for AD, since available drugs provide only palliative effects or merely delay the onset of symptoms, which underscores the importance of early diagnosis of this disorder [4]. Associated pathological alterations can be observed in the brains of patients with mild cognitive impairment (MCI), a prodromal stage of AD in which cognitive symptoms do not yet interfere significantly with daily life. Moreover, molecular pathology of AD has been detected in the brain as early as 20 years before the appearance of clinical symptoms, in what is known as the preclinical stage of the disease [5,6]. Most AD cases occur in individuals over 65 years of age and have a multifactorial origin, associated with lifestyle- or age-related risk factors, although some cases are primarily genetic in origin [7,8]. AD is characterized by atrophy of the hippocampus and the entorhinal cortex, which are key structures involved in memory formation, consolidation and processing of sensory information. This neurodegeneration leads to memory deficits and progressive cognitive decline, the severity of which is used to classify the disease into three stages: mild (or early), moderate (or intermediate) and severe (or late) [9,10]. This decline, which reflects disease progression, can be quantified using various tests, such as the Mini-Mental State Examination (MMSE) and the Montreal Cognitive Assessment (MoCA) test [11].

At the molecular level, AD pathology begins with the accumulation of amyloid-beta (Aβ) peptide in the brain, which aggregates and deposits extracellularly in the form of senile plaques. Subsequently, hyperphosphorylation and accumulation of the so-called tau protein occurs, whose primary function is to stabilize the cytoskeleton [5,12]. Once tau becomes hyperphosphorylated (pTau), it loses its affinity for microtubules and becomes more prone to aggregation, forming neurofibrillary tangles that accumulate intracellularly and disrupt neuronal function, including synaptic activity, axonal transport and protein degradation pathways [13]. Additionally, the accumulation of Aβ and pTau activates microglia, triggering inflammation which, if sustained chronically, may result in neurodegeneration and cell death, both neuronal and glial [14].

AD can be diagnosed on the basis of various biomarkers, which are categorized into three main groups constituting the AT(N) framework: (1) biomarkers of amyloid (A) pathology, such as Aβ levels in the cerebrospinal fluid (CSF) and the presence of senile plaques in the brain, the latter detected by positron emission tomography (PET); (2) biomarkers of tau (T) pathology, such as pTau levels in the CSF; and (3) biomarkers of neurodegeneration or neuronal injury (N), such as total Tau (tTau) levels in the CSF, brain glucose hypometabolism measured by PET, or cortical atrophy detected by magnetic resonance imaging (MRI) [15]. In addition, other techniques can be used to monitor AD, including microRNA (miRNA) sequencing for the identification of novel biomarkers, MRI and the application of artificial intelligence (AI) technologies to improve diagnostic accuracy through the analysis of specific biomarkers [16,17,18]. AI has also been explored for the diagnosis of glaucoma, and the US Food and Drug Administration has approved an AI-based system for diagnosing diabetic retinopathy [19,20].

The retina is a constituent part of the central nervous system (CNS), and as such, shares many structural and functional features with the brain. As a result, the retina is also affected by neurodegenerative diseases that impact the brain in both humans and animal models, such as Parkinson disease (PD), AD, multiple sclerosis and ictus, to name a few [21,22,23,24,25]. Pathological alterations associated with AD are detectable not only in the retina, but also in other ocular components, such as the vitreous humor and the choroid, as well as in parameters related to visual function [21,26]. This review addresses the retinal and ocular alterations reported to date that may hold potential or demonstrated utility for AD diagnosis and the monitoring of its progression in the brain.

## 2. Molecular Biomarkers

A large number of molecular alterations associated with AD have been identified in various ocular components, particularly in the retina, as summarized in Table 1. In this context, western blotting and immunohistochemical analyses have revealed elevated levels of Aβ peptide in the postmortem retinas of AD mouse models. Moreover, Aβ detection using curcumin staining was achieved prior to the appearance of detectable amyloid deposits in the brain [27,28]. Also, Aβ plaques have been identified in the retinas of patients with AD, predominantly in the inner retinal layers [29]. This retinal Aβ deposition correlates with Aβ plaque formation in the brain, specifically within the visual cortex, and is associated with AD progression [29]. Aβ peptide accumulation has also been observed in the retinas of patients with age-related macular degeneration and glaucoma, mainly being found in the central retina in these cases, in contrast with its distribution in the peripheral retina of AD patients [25,29].

To facilitate diagnosis and prognosis of AD based on retinal Aβ burden, non-invasive techniques, such as scanning laser ophthalmoscopy (SLO), are employed to quantify retinal Aβ in vivo. Using this approach, amyloid accumulation has been detected in the retinas of AD patients following administration of curcumin, a naturally fluorescent compound with high affinity for the Aβ peptide [29,30]. Additionally, confocal SLO has been used to visualize retinal autofluorescence, revealing a correlation between retinal Aβ deposition and cerebral amyloid load, the former quantified on the basis of the area of autofluorescent bodies containing fibrillar Aβ, predominantly localized to the inner plexiform layer (IPL), and the latter measured by PET [31].

Hyperspectral imaging (HSI) of the retina has revealed an increased reflectance in AD patients compared to healthy controls, which interestingly correlated with cerebral Aβ peptide levels [32,33]. Consequently, HSI has been proposed as a valuable tool for monitoring disease progression and potentially detecting cerebral Aβ aggregation. This technique has thus shown promising utility in distinguishing AD cases from healthy controls. However, other non-specific factors, such as the presence of pTau or inflammation, may also contribute to reflectance changes. Additionally, the use of a small validation cohort raises the need for further research to establish the utility of HSI for AD diagnosis in clinical practice [32,33].

Elevated levels of tTau and pTau have been detected in the retinas of AD patients and transgenic AD-model mice carrying human mutations in genes encoding tau, amyloid precursor protein (APP) and presenilin-1, all of which are well known to be involved in the pathogenesis of AD [12,13]. Notably, retinal tau alterations appear to precede brain pathology in these model mice by several months [38], and tau isoforms phosphorylated at residues Ser202/Thr205 (pTau^S202/T205^) and/or Thr217 (pTau^T217^) correlate in humans with their presence in the cerebral cortex and hippocampus, and are associated with AD progression [37]. Increased levels of tau phosphorylated at Ser214, Thr231, Ser396 and Ser404, as well as tau citrullinated at Arg209 and oligomeric tau species, have been reported in the retinas of patients suffering MCI or AD compared to healthy controls [34]. Moreover, an increased number of retinal ganglion cells (RGCs) positive for pTau^S396^ or oligomeric tau have been found in MCI and AD patients, with RGC counts correlating with cerebral disease progression [35]. Given the specificity to tauopathies such as AD of alterations in the levels of these particular tau isoforms, their assessment in the retina shows promise for differential diagnosis [35]. Recently, the anatomical distribution of various tau isoforms, including pTau^S202/T205^ and pTau^T217^, has been examined across retinal layers in AD patients, primary age-related tauopathy cases, and healthy controls. A four-stage progression model of retinal tau pathology has been established, starting with absence of pTau^S202/T205^ and followed by sequential accumulation of this isoform in the outer plexiform layer, inner nuclear layer and IPL [36]. This progression correlated with age and visual deterioration, and was associated with AD, but not with the progression of cerebral tau pathology [36].

Amyloid-beta isoforms Aβ40 and Aβ42 have been detected in the lenses of AD patients at levels comparable to those in the cerebral cortex, this promoting aggregation of lens proteins such as αB-crystallin [48]. However, other studies have failed to detect deposits of Aβ, tau or other, additional molecular markers in the lenses of AD individuals [49]. In order to discriminate AD patients from cognitively normal individuals, a method has been developed to detect fluorescence signals from the ligand aftobetin hydrochloride bound to Aβ in the supranuclear region of the lens, where protein aggregation is most pronounced, achieving high sensitivity and specificity [40]. Average fluorescence signals in AD cases were approximately twice those in controls and correlated with PET measures of cerebral amyloid load using the ^18^F-florbetapir radiotracer [39,40].

In AD mouse models, Aβ42 and Aβ40 have been detected in the vitreous and aqueous humors, with cognitive function assessed by the MMSE test correlating with levels of Aβ and tTau in the vitreous humor [41,50]. Recently, elevated tTau levels have also been detected in the vitreous humor of AD patients, with a negative correlation found for tTau and a positive correlation detected for the pTau^S231^ isoform relative to their corresponding cortical levels [42]. Additionally, the Aβ peptide can be transported from the brain to the eye via the CSF surrounding the optic nerve and also through aquaporin-4 channels in glial cells, namely perivascular astrocytes surrounding cerebral and ocular blood vessels, and Müller cells, which contain aquaporin-4 channels mainly in their endfeet extending to the inner limiting membrane (ILM) and ensheathing retinal blood vessels. These channels participate in the ocular glymphatic clearance system responsible for the removal of excess intraocular fluid and metabolic waste from the retina [51,52]. This system accumulates Aβ in both AD patients and the 5xFAD mouse model overexpressing mutant human APP and PS1 proteins associated with familial AD [52]. Furthermore, a correlation between the volume of Aβ plaques in the eyeball and cerebral amyloid burden was demonstrated in 5xFAD mice using 3D light-sheet fluorescence microscopy [43].

In recent years, molecular biomarker analysis in tear fluid for the diagnosis and prognosis of several neurological and retinal diseases—including AD, PD, multiple sclerosis and glaucoma—has gained significant interest due to its accessibility and CNS disease associations [53,54,55]. Reduced Aβ42 levels have been reported in the tear fluid from MCI and AD patients, correlating with their cognitive decline evaluated by psychometric tests such as the MMSE and AD rating scales, this highlighting the potential of tear-based biomarkers for AD diagnosis [44]. Tear Aβ concentrations in healthy individuals can be up to 10-fold higher than serum levels, this underscoring the utility of tear fluid for less invasive and cost-effective AD diagnosis [53,56]. Conversely, elevated tTau levels have been found in tear fluid from AD patients compared to MCI patients and healthy controls, as well as in individuals with neurodegeneration characterized by high tau concentrations in the CSF [45]. A negative correlation between tTau levels in tear fluid and Aβ42 levels in the CSF—typically decreased in AD patients—supports the use of tear biomarkers [45,57]. Proteomic studies have also identified differentially expressed proteins in the tear fluid of AD patients relative to controls, with four proteins showing a strong potential as biomarkers for disease discrimination, namely lipocalin-1 (LCN1), lactoferrin, lysozyme C and Pro-rich protein 4 (PRR4), with key roles in ocular homeostasis, innate local immunity and protection against oxidative stress and inflammation [47]. Additionally, 12 other proteins, mostly involved in metabolic processes such as protein biosynthesis, folding and degradation (proteostasis), and mRNA stability (e.g., eIF4E, NARS1, PSMD3 and ERP29), were uniquely present in AD tears [46]. Finally, miRNA expression analysis has revealed significantly elevated levels in AD tear fluid of miRNA-200b-5p, a molecule known to counteract Aβ toxicity [46,58].

## 3. Structural Biomarkers

A number of structural alterations have been described to date in various ocular components and retinal layers that are associated with AD, as detailed below and summarized in Table 2. In AD patients, a significant degeneration of RGCs has been observed, attributed to intracellular accumulation of Aβ peptide within their cytoplasm, whereas in APP/PS1 mice, Aβ accumulation has been reported to induce apoptosis in this retinal neuronal type [59,60]. The loss of neuronal and axonal densities in the retina may be reflected in the thickness of the retinal layers where these structures are located, such as the IPL, ganglion cell layer (GCL) and nerve fiber layer (NFL), which contain the dendrites, somata and axons of RGCs, respectively [61].

Several meta-analyses have demonstrated that patients with MCI or AD exhibit characteristic morphological changes in the retina, as revealed by non-invasive assessment of retinal layer thickness and topography using optical coherence tomography (OCT). These studies have revealed that a significant thinning of the NFL and combined IPL+GCL layers takes place in such patients compared to healthy controls, as well as a reduction in retinal thickness at the fovea [62,63,64,65,66]. Further thinning of various retinal layers, particularly the NFL, has also been reported in patients with multiple sclerosis or PD, as well as in individuals with certain psychiatric disorders, such as schizophrenia or anorexia [67,68]. Additionally, it has been shown that retinal features observed from OCT and fundus imaging were both associated with brain measures obtained by magnetic resonance imaging (MRI) [69]. This highlights the predictive potential of retinal structural analysis in the assessment of cognitive decline and neurodegenerative diseases such as AD and PD [69].

In individuals with preclinical AD, a thinning of the NFL in the macular area has been observed to precede structural changes in the peripapillary region surrounding the optic disc [70]. This early macular thinning correlates with cerebral Aβ peptide deposition, as quantified by PET imaging using ^18^F-florbetapir as the radiotracer [70], which binds to β-amyloid. Moreover, NFL thickness has been correlated with progression in episodic memory performance, that is, memory related to events and experiences, as well as with clinical dementia ratings and scores on the Alzheimer Disease Assessment Scale-Cognitive subscale (ADAS-Cog) [71,72]. However, other studies have not found correlations between NFL thickness and MMSE scores, possibly due to the characteristics of this test and of the cohorts studied [72,73]. Recently, a positive correlation has been reported between hippocampal volume and the thickness of peripapillary NFL [74], supporting the potential use of the latter as a valid parameter for non-invasive monitoring of AD progression. Similarly, several studies have reported an association between the thicknesses of different retinal layers quantified by OCT, such as the NFL and IPL+GCL, and various brain structural parameters measured by MRI, including hippocampal volume and total brain volume [75]. An algorithm has thus been developed aimed at extracting various quantitative parameters from each retinal layer based on OCT images, collectively termed ‘spatial intensity correlation features’, which were found to be altered in individuals with MCI or AD compared to healthy controls [76].

**Table 2:** Structural ocular biomarkers for the diagnosis and prognosis of Alzheimer disease.

Ocular Structure or Tissue	Alteration	Utility
Retina	↓ Total macular thickness and volume [62,65,66,73,77,78,79,80]	Prognosis [75,77,78,81]
	↓ NFL and IPL+GCL thickness [62,63,64,65,66,70,71,72,73,76,77,78,79,80,82]	Prognosis [70,71,72,75,78]
	↑, ↓ Quantitative OCT imaging parameters [76]	Diagnosis and prognosis [76]
	↓ SCP and DCP vascular density [63,83,84,85,86,87,88]	Prognosis [85,86,87,88]
	↓ Vessel fractal dimension and venular caliber [66,89,90]	Prognosis [90,91]
	↓ Blood flow [91,92]	Prognosis [91]
	↑ FAZ area [63,83,84,86,88,89,93]	Prognosis [88]
	Retinal and fundus imaging via AI models (OCT-A+AI) [78,94,95,96,97,98,99]	Diagnosis [78,94,95,96,97,98,99]
Optic nerve	↑ Cup-to-disc ratio [100]	Prognosis [100]
Choroid	↓ Thickness [65,66]	Diagnosis and prognosis [101]
	↓ Choriocapillaris microvascular density [101]	
Cornea	↓ Nerve fiber density, branching and length [102,103]	Diagnosis [102,103] and prognosis [102]

Arrows pointing upwards or downwards indicate increase or decrease, respectively, in the parameters indicated.

Furthermore, these features were used to build a multivariable model capable of discriminating between MCI or AD patients and healthy controls with higher specificity and sensitivity than models based solely on the thickness of retinal layers [76]. Additionally, total macular thickness has been correlated with total and parietal cortical atrophy quantified by MRI in both AD patients and healthy controls [81]. A correlation was also found between macular thickness and MMSE scores in AD patients [77]. Moreover, the thickness of the photoreceptor outer segment layer has been associated with total brain volume, suggesting that the retina may reflect cerebral alterations independently of Aβ toxicity [104]. Finally, degeneration of the optic nerve (composed of RGC axons) has been observed in AD patients, with the optic cup-to-disc ratio correlating with ADAS-Cog score, the latter being a measure of disease severity [82,100].

Retinal vasculature may reflect the status of cerebral blood vessels, given their similarities in both structural and pathological changes associated with conditions such as hypertension and diabetes [75,105]. Age-related increases in Aβ deposition have been observed in vessels of the cerebral cortex, retina and choroid of both wild-type and AD model mice, potentially impairing blood perfusion and cellular metabolism in these regions and contributing to retinal inflammation [59,106,107]. As a consequence, those alterations could lead to morphological changes in the retinal vasculature that can be detected using OCT angiography (OCT-A). Furthermore, the reciprocal effects and interconnection between vascular alterations and neurodegeneration have been studied in the brain, highlighting the complex etiology of AD, although the main underlying mechanisms in the retina are unknown [108]. Several meta-analyses using OCT angiography (OCT-A) data have demonstrated a significant reduction in the vascular density of the superficial and deep capillary plexi (SCP and DCP, respectively) in the retinas of patients with MCI or AD, as compared with cognitively healthy controls [63,83,84]. Conversely, increased retinal vascular density has been reported in presymptomatic individuals exhibiting cerebral Aβ deposition detected by PET imaging [85], with this vascular parameter correlating with MMSE scores [86,88]. This discrepancy could be explained by the occurrence of both protein aggregation and neuroinflammation in the retina at a presymptomatic stage, which might lead to the increase in retinal vascular density observed, followed by its subsequent decrease caused by neurodegeneration and vascular atrophy [109]. Furthermore, vascular density in the SCP was found to correlate with Montreal Cognitive Assessment (MoCA) scores, highlighting the potential utility of OCT-A for monitoring AD progression in the brain [87]. Additionally, decreases in retinal venular caliber and in fractal dimension of retinal vessels have been found, the latter being associated with cognitive decline [66,90,91]. In particular, a reduced fractal dimension of the superficial vascular complex—comprising vessels located in the IPL, GCL and NFL—has been observed in cognitively healthy individuals with pathological profiles of AD retinal vasculature biomarkers [89].

Retinal blood flow has been shown to be reduced in MCI and AD patients compared to controls, and to decrease with age and dementia severity, as quantified non-invasively by laser Doppler flowmetry [92]. Moreover, multiple studies and meta-analyses have reported an enlargement of the foveal avascular zone (FAZ) area in patients with MCI or AD and, importantly, also in individuals in the preclinical stage of AD, i.e., prior to the onset of overt cognitive symptoms [63,83,84,86,89,93]. The FAZ area has also been correlated with MMSE cognitive scores, although significant interindividual variability in this area may limit its clinical use unless complemented by additional ocular biometry assessments [88,110,111]. In patients with MCI or AD, OCT has revealed choroidal thinning, as it occurs in PD patients and healthy individuals as they age [65,66,112,113]. Furthermore, an altered microvascular density in the choriocapillaris layer—the innermost stratum of the choroid—has been detected in AD patients by means of OCT-A quantitation [101]. This parameter correlated with serum biomarkers of the disease, such as Aβ42 and Aβ40 levels, showing a potential for discriminating early-onset AD patients from healthy controls [101].

Artificial intelligence (AI) has shown promise in discriminating patients with MCI or AD from healthy controls using brain MRI and PET imaging, as demonstrated in systematic reviews and studies of ocular biomarkers, thus supporting that similar AI-based approaches could be extended to retinal and fundus imaging [17,114]. In this context, several AI models trained on OCT retinal images have been developed, using machine learning, which are capable of distinguishing AD patients from cognitively healthy individuals with high accuracy [78,94]. Nevertheless, larger and more heterogeneous cohorts would be needed to validate those models and study their diagnostic potential. Similarly, AI models trained on fundus images have achieved a high sensitivity and specificity in diagnosing MCI and AD, and a deep learning model has even identified accurately the presence or absence of cerebral Aβ deposition in patients and controls, respectively [95,96,97]. The incorporation of quantitative retinal biometric data and patient demographics, such as age, sex and years of education, into retinal image-based algorithms has improved the discrimination between healthy individuals and those with MCI or AD [98,99]. However, and despite its diagnostic utility, AI has not yet succeeded in predicting cognitive decline in AD patients even when trained with deep learning on large datasets of fundus images combined with patients’ demographic data and clinical information [115]. The lack of large, heterogeneous datasets may limit the accuracy of existing AI models for AD detection, along with other factors such as poor image quality and imbalanced datasets, which could also hinder the short-term applicability of these diagnostic approaches [17,116].

In addition to the retina, corneal nerve fibers have been studied via confocal microscopy, revealing age-related alterations as well as disease-specific changes in patients exhibiting particular disorders such as multiple sclerosis and diabetes [117,118,119]. In this fashion, decreased corneal nerve fiber density, branching, and length have been reported in MCI and AD patients compared to cognitively healthy individuals, with these reductions correlating with cognitive impairment evaluated by the MoCA test [102,103]. These parameters enabled the discrimination of AD cases from healthy controls with an accuracy comparable to that of the medial temporal lobe atrophy index, a biomarker commonly used for diagnostic support, and even surpassed this index in distinguishing MCI cases [103].

## 4. Functional Biomarkers

Several studies have identified a number of visual and functional alterations in various ocular components that are associated with AD, detectable through non-invasive techniques. These findings are detailed below and summarized in Table 3.

Pattern electroretinography (PERG) is a non-invasive technique that records the retina’s electrical responses to patterned light stimuli, primarily reflecting the functional integrity of photoreceptors, as well as of RGCs and their axons [120]. In AD patients, even at early stages, PERG has revealed reduced amplitudes and increased implicit times of the N35, P50, and N95 waves compared to healthy individuals [121,122,123]. Moreover, specific quantifiable parameters obtained through electroretinography (ERG) have proven useful in classifying cognitively healthy individuals based on the Aβ42/tau ratio in their CSF [124]. Among these parameters, the amplitude of the photopic negative response has shown potential as an early diagnostic marker for AD [124]. Notably, abnormal retinal electrical responses measured by PERG have also been reported in patients with other neurological conditions, such as PD, multiple sclerosis, and major depressive disorder [67,125].

Visual acuity has also been reported to be reduced in individuals with MCI or AD compared to healthy controls, showing significant correlations with MMSE scores and an increased risk of developing AD in individuals over 65 years of age [79,126,127]. Similarly, deficits in contrast sensitivity have been observed in MCI and AD patients, also correlating with MMSE scores and with performance on the California Verbal Learning Test, a measure of memory function. Additionally, contrast sensitivity threshold values have been demonstrated to exhibit good discriminatory power between MCI patients and cognitively healthy individuals [79,80,126,128,129]. Moreover, patients with AD and without a history of color blindness have been found to exhibit impaired color discrimination, which is associated with cognitive decline [79,126,130,131,132]. In this context, the number of errors in various color discrimination tests has demonstrated diagnostic potential for distinguishing MCI and AD patients from cognitively healthy individuals [79,126,131]. Furthermore, poorer color discrimination has been detected in individuals with signs of neurodegeneration in the brain, measured by PET, which was not observed in those with signs of brain Aβ deposition [132]. In addition, a diminished ability to perceive the direction and speed of moving objects has also been documented in AD patients, correlating with dementia severity and cognitive impairment, similarly to changes observed in cognitively healthy older adults [133,134,135].

A recent meta-analysis has highlighted increased saccadic latency and a higher rate of directional errors in anti-saccade tasks in MCI and AD patients compared to healthy controls [136]. Several studies have also reported correlations between these oculomotor parameters and measures of cognitive decline, particularly episodic memory impairment(s) [136,137,138,139]. Based on these findings, an algorithm was developed able to accurately discriminate MCI and AD patients from cognitively healthy individuals with high sensitivity [140]. Oculomotor alterations could also be explained as a consequence of the degeneration and dysfunction of motor cortical neurons, as shown in the AD mouse model 5xFAD, in which Aβ accumulation has been detected in the primary motor cortex and other cortical areas [141].

Additionally, abnormalities in the pupillary light reflex—i.e., the constriction or dilation of the pupil in response to light—have been observed in patients with AD. Specifically, a significant reduction in both the velocity and acceleration of pupillary constriction has been reported. These parameters, when combined with age and the presence of the *APOE*-ε4 allele (in homozygosity or heterozygosity), have enabled accurate discrimination between AD patients and cognitively healthy individuals [142,143]. Recently, a relationship has been found between the functional connectivity of the neural network regulating pupil diameter and both visuospatial and memory functions [144]. This network involves the locus coeruleus, whose early degeneration and dysfunction are considered key biomarkers of AD progression. These findings have underscored the prognostic potential of pupillometric analysis in the context of AD [145].

**Table 3:** Functional ocular biomarkers for the diagnosis and prognosis of Alzheimer disease.

Ocular Function	Alteration	Utility
Retinal electrical activity (photoreceptors and RGCs)	↓ Amplitude and ↑ implicit time of PERG waves (N35, P50 and N95) [121,122,123]	Diagnosis [124]
	↓ Amplitude of photopic negative response [124]	
Visual acuity	↓ Eyes’ ability to distinguish fine details at a distance [79,126]	Diagnosis and prognosis [126,127]
Contrast sensitivity (RGCs and brain)	↓ Contrast discrimination across spatial frequencies [79,80,126,128,129]	Diagnosis [128] and prognosis [126,128,129]
Color vision (cone photoreceptors)	↑ Errors in discrimination of light wavelengths [79,80,126,130,131,132]	Diagnosis [131] and prognosis [126,130,131]
Motion perception (retina and brain)	↓ Ability to perceive direction and speed of motion [133,134,135]	Prognosis [135]
Saccadic eye movements (brain)	↑ Latency of saccade initiation and ↓ anti-saccade control [136,137,138,139]	Diagnosis [140] and prognosis [138,139]
Pupillary light reflex (brain)	Pupillary constriction ↓ amplitude and ↑ latency [142]	Diagnosis and prognosis [143]

Arrows pointing upwards or downwards indicate increase or decrease in levels, respectively, of the parameters indicated.

## 5. Current Limitations and Challenges

Although the potential of the eye and the retina as sources of potential molecular, structural and functional biomarkers of AD and MCI is undeniable, there are several limitations that hinder their applicability for clinical diagnosis and prognosis, as well as the interpretation of some of the findings brought to light by the studies reviewed in this work. Firstly, it is worth mentioning that data from different studies and laboratories can differ considerably depending, among other factors, on the ages, characteristics and ethnicity of the patients analyzed or, considering AD transgenic mice, the strain and sex of the individuals used, thus posing difficulty in their comparison and bringing up reproducibility issues. Additionally, results from existing AD animal models could not be fully extrapolatable to humans, as they may not represent the multifactorial nature of AD pathology and etiology. The use of different imaging devices, histological techniques and analytical methods may influence variability between studies, which highlights the importance of methodology standardization and validation of data from different centers, in order to reduce the variability of results. Additionally, the diagnostic criteria used for AD and, in particular, MCI are a critical factor that may contribute to inconsistent findings across studies. This is especially relevant for MCI, as it represents a heterogeneous condition characterized by variable clinical trajectories and rates of progression. Consequently, differences in patient classification can lead to substantial variability in the data obtained. Therefore, standardized diagnostic guidelines, such as those proposed by the National Institute on Aging–Alzheimer’s Association, should be consistently applied to classify individuals, thereby enhancing comparability among studies and improving diagnostic reliability.

On the other hand, several neurodegenerative processes and retinal alterations associated with AD overlap with those observed in other dementias and retinal pathologies, such as glaucoma or AMD. As a result, some of the ocular biomarkers discussed in this review lack disease specificity, thereby limiting their utility for differential diagnosis. For instance, retinal Aβ deposition and thinning of particular layers have also been reported in patients with AMD or glaucoma. Nevertheless, these alterations appear to exhibit different spatial distribution patterns, which may provide a basis for distinguishing between these pathologies. Moreover, aging and age-related changes share various similarities with AD, as they are also associated with Aβ accumulation, oxidative damage and inflammation and induce similar morphological alterations. It should also be noted that different conditions may coexist in the same individual, and may even occur with a higher prevalence among patients with AD, thereby complicating the diagnosis and highlighting the need for more specific biomarkers. Furthermore, the combination of different non-invasive retinal biomarkers, images, visual function tests and various clinical data, such as age and sex, could significantly enhance the diagnostic specificity and sensitivity.

## 6. Conclusions

**Figure 1:**
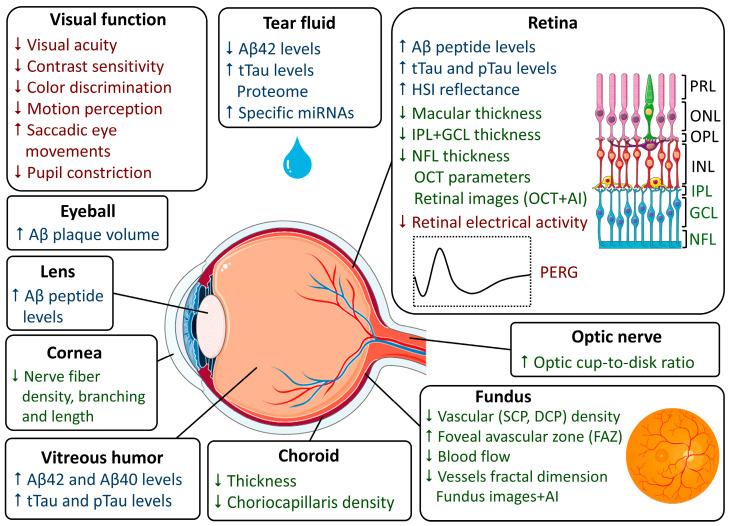
Molecular, structural and functional ocular biomarkers associated with Alzheimer disease. The scheme compiles the alterations detectable in various components of the eye, pertaining both to the anterior segment (such as the cornea and lens) and the posterior segment (such as the choroid, vitreous humor, optic nerve and retina, including its vasculature). Molecular, structural and functional ocular biomarkers are indicated in blue, green and red, respectively. Protein levels of Aβ and tau in different tissues and fluids can be quantified by western blotting or immunohistochemistry, whereas their presence can be assessed qualitatively or semi-quantitatively through non-invasive techniques such as scanning laser ophthalmoscopy (SLO) or retinal hyperspectral imaging (HSI). Such structural alterations can be detected using imaging methods such as optical coherence tomography (OCT) coupled to artificial intelligence (OCT+AI), fundus photography or confocal laser scanning microscopy (for corneal examination). Those structural alterations include changes in the superficial (SCP) and deep (DCP) capillary plexi, as well as in the innermost retinal layers, namely inner plexiform layer (IPL), ganglion cell layer (GCL) and nerve fiber layer (NFL). [For other retinal-layer abbreviations, see text] OCT parameters derived from an algorithm include three intensity spatial correlation features: angular second matrix, correlation, and homogeneity. Lastly, a range of specialized techniques are available to measure parameters indicative of visual function, such as pattern electroretinography (PERG), or to monitor electrical impulse transmission, together with a variety of visual functional tests designed to evaluate visual acuity, color discrimination, contrast sensitivity and pupillary light reflex, among others. Images were provided by Servier Medical Art (https://smart.servier.com), licensed under CC BY 4.0 (https://creativecommons.org/licenses/by/4.0/). Abbreviations: PRL, photoreceptor layer; ONL, outer nuclear layer; OPL, outer plexiform layer; INL, inner nuclear layer. For other abbbreviations, see table below.

AD is widely recognized as a neurodegenerative disorder that primarily affects the brain. However, growing evidence indicates that AD is also associated with a variety of pathological alterations in ocular tissues and structures—particularly in the retina—several of them correlating with the progression in the brain, which can be detected at early stages through non-invasive imaging techniques and are compiled in Figure 1. Yet, most of the underlying mechanisms responsible for such ocular abnormalities remain poorly understood, representing a promising avenue for future research.

To effectively translate current findings into clinical practice, further studies are needed to evaluate the feasibility and diagnostic utility of ocular assessment techniques for AD, as well as their integration with established neurodiagnostic methods. Current evidence suggests that the eye, and particularly the retina, may represent a promising source of non-invasive biomarkers for the early and predictive diagnosis of MCI and AD. These findings support the use of ocular biomarkers as complementary tools to current diagnostic approaches and as a foundation for the development of novel strategies aimed at accurately predicting disease progression.

## Figures and Tables

**Table 1:** Molecular ocular biomarkers for the diagnosis and prognosis of Alzheimer disease.

Ocular Structure, Tissue or Fluid	Alteration	Utility
Retina	↑ Aβ peptide levels and plaques [28,29,30,31]	Prognosis [28,29]
	↑ HSI reflectance [32,33]	Diagnosis and prognosis [33]
	↑ tTau and pTau levels [34,35,36,37,38]	Prognosis [34,35,36,37]
Lens	↑ Aβ peptide levels [39,40]	Diagnosis and prognosis [40]
Vitreous humor	↑ Aβ42 and Aβ40 levels [41]	Prognosis [41]
	↑ tTau and pTau231 levels [41,42]	Prognosis [41,42]
Eyeball	↑ Aβ plaque volume [43]	Prognosis [43]
Tear fluid	↓ Aβ42 levels [44]	Diagnosis and prognosis [44]
	↑ tTau levels [45]	Prognosis [45]
	↑, ↓ Differential protein expression [46,47]	Diagnosis [47]
	↑ miRNA-200b-5p levels [46]	

Arrows pointing upwards or downwards indicate increase or decrease in levels, respectively.

## Data Availability

No new data were created or analyzed in this study.

## Abbreviations

Aβ	Amyloid-beta peptide
AD	Alzheimer disease
ADAS-Cog	AD Assessment Scale-Cognitive Subscale
AI	Artificial intelligence
CNS	Central nervous system
CSF	Cerebrospinal fluid
DCP	Deep capillary plexus
ERG	Electroretinography
FAZ	Foveal avascular zone
GCL	Ganglion cell layer
HSI	Hyperspectral imaging
IPL	Inner plexiform layer
MCI	Mild cognitive impairment
miRNA	microRNA
MMSE	Mini-Mental State Examination
MoCA	Montreal Cognitive Assessment
MRI	Magnetic resonance imaging
NFL	Nerve fiber layer
OCT	Optical coherence tomography
OCT-A	OCT angiography
PD	Parkinson disease
PERG	Pattern ERG
PET	Positron emission tomography
pTau	Phosphorylated tau
RGC	Retinal ganglion cell
SCP	Superficial capillary plexus
SLO	Scanning laser ophthalmoscopy
tTau	Total tau
